# Exploring the effects of coexisting amyloid in subcortical vascular cognitive impairment

**DOI:** 10.1186/s12883-015-0459-1

**Published:** 2015-10-12

**Authors:** Elizabeth Dao, Ging-Yuek Robin Hsiung, Vesna Sossi, Claudia Jacova, Roger Tam, Katie Dinelle, John R. Best, Teresa Liu-Ambrose

**Affiliations:** Djavad Mowafaghian Centre for Brain Health, University of British Columbia, 2215 Wesbrook Mall, Vancouver, BC V6S 0A9 Canada; Department of Medicine, University of British Columbia, 2211 Wesbrook Mall, Vancouver, BC V6T 2B5 Canada; Department of Physics and Astronomy, University of British Columbia, 6224 Agricultural Road, Vancouver, BC V6T 1Z1 Canada; School of Professional Psychology, Pacific University, 190 SE 8th Avenue, Hillsboro, OR 97123 USA; Department of Radiology, University of British Columbia, 3350-950 W 10th Avenue, Vancouver, BC V5Z 1 M9 Canada; MS/MRI Research Group, University of British Columbia, 2215 Wesbrook Mall, Vancouver, BC V6S 0A9 Canada; UBC PET, Brain Research Centre, 2211 Westboork Mall, Vancouver, BC V6T 2B5 Canada; Department of Physical Therapy, University of British Columbia, 212-2177 Wesbrook Mall, Vancouver, BC V6T 1Z3 Canada

**Keywords:** Vascular cognitive impairment, Amyloid, Mixed dementia, Cognitive function

## Abstract

**Background:**

Mixed pathology, particularly Alzheimer’s disease with cerebrovascular lesions, is reported as the second most common cause of dementia. Research on mixed dementia typically includes people with a primary AD diagnosis and hence, little is known about the effects of co-existing amyloid pathology in people with vascular cognitive impairment (VCI). The purpose of this study was to understand whether individual differences in amyloid pathology might explain variations in cognitive impairment among individuals with clinical subcortical VCI (SVCI).

**Methods:**

Twenty-two participants with SVCI completed an ^11^C Pittsburgh compound B (PIB) position emission tomography (PET) scan to quantify global amyloid deposition. Cognitive function was measured using: 1) MOCA; 2) ADAS-Cog; 3) EXIT-25; and 4) specific executive processes including a) Digits Forward and Backwards Test, b) Stroop-Colour Word Test, and c) Trail Making Test. To assess the effect of amyloid deposition on cognitive function we conducted Pearson bivariate correlations to determine which cognitive measures to include in our regression models. Cognitive variables that were significantly correlated with PIB retention values were entered in a hierarchical multiple linear regression analysis to determine the unique effect of amyloid on cognitive function. We controlled for age, education, and ApoE ε4 status.

**Results:**

Bivariate correlation results showed that PIB binding was significantly correlated with ADAS-Cog (p < 0.01) and MOCA (p < 0.01); increased PIB binding was associated with worse cognitive function on both cognitive measures. PIB binding was not significantly correlated with the EXIT-25 or with specific executive processes (p > 0.05).

Regression analyses controlling for age, education, and ApoE ε4 status indicated an independent association between PIB retention and the ADAS-Cog (adjusted R-square change of 15.0 %, Sig F Change = 0.03). PIB retention was also independently associated with MOCA scores (adjusted R-Square Change of 27.0 %, Sig F Change = 0.02).

**Conclusion:**

We found that increased global amyloid deposition was significantly associated with greater memory and executive dysfunctions as measured by the ADAS-Cog and MOCA. Our findings point to the important role of co-existing amyloid deposition for cognitive function in those with a primary SVCI diagnosis. As such, therapeutic approaches targeting SVCI must consider the potential role of amyloid for the optimal care of those with mixed dementia.

**Trial registration:**

NCT01027858

## Background

The world’s population is rapidly aging and the total number of people living with dementia is projected to increase globally from 24.3 million in 2001 to 81.1 million in 2040 [1]. Alzheimer’s disease (AD) and vascular cognitive impairment (VCI) are the two most common causes of cognitive dysfunction [2]. AD is a neurodegenerative disease characterized by amyloid-beta (Aβ) plaques and neurofibrillary tangles (NFT) [3]. Individuals with AD often present with impaired episodic memory, defined as the conscious retrieval of autobiographical events [3, 4]. Vascular cognitive impairment can be associated with both large vessel disease and small vessel disease [5]; this study will focus on those with subcortical VCI (SVCI) as this group is suggested to be a more homogenous group of patients that are expected to show greater predictability in their clinical picture, natural history, outcome, and treatment response [6]. SVCI is caused by small vessel damage that is typically associated with chronic and diffuse hypoperfusion causing cerebral white matter lesions (WML) and lacunes [7]. People with SVCI display relatively intact episodic memory, but show impairments on measures of executive functions, defined as higher-order cognitive processes underpinning goal directed behaviors [8].

AD and SVCI are often reported as two distinct diseases in epidemiological studies; however, evidence from neuropathological studies indicate a high rate of mixed AD-vascular pathology, generally referred to as “mixed dementia”. Mixed pathology is present in approximately half of all clinically diagnosed AD cases [9–13], including participants of AD clinical trials who were extensively screened for pure AD [14]. An autopsy study reported AD with cerebrovascular lesions to be the second most common pathology after AD [15]; thus, mixed pathology may often be the rule rather than the exception in clinical diagnosis. Recently, efforts were made in understanding the manifestation of AD with cerebrovascular disease [16]. For example, at the early AD pathology stage of entorhinal cortical involvement–which is generally clinically asymptomatic–the presence of cerebrovascular lesions is associated with cognitive impairment. This suggests that cerebrovascular lesions may lower the threshold for dementia [17]. In addition, among those with AD, the presence of ischemic lesions is associated with a greater degree of cognitive deficits compared with pure AD pathology. Overall, it is hypothesized that vascular lesions may magnify the effect of mild AD pathology, result in more severe cognitive impairment, and accelerate disease progression [18]. Currently, much of our knowledge on mixed dementia stems from the perspective of a primary AD diagnosis and hence, little is known on the effects of secondary AD pathology in a primary SVCI diagnosis. Specifically, it is unclear how co-existing amyloid pathology may affect cognitive function in people with a primary clinical SVCI diagnosis.

To investigate cognitive function in mixed pathology it is important to include cognitive domains associated with both SVCI and AD to understand the full spectrum of cognitive impairment. There is consensus that cognitive measures such as the Alzheimer’s Disease Assessment Scale-cognitive subscale (ADAS-Cog) [19], the Executive Interview Test (EXIT-25) [20], and the Montreal Cognitive Assessment (MOCA) [21] should be included for an optimal assessment battery in AD and VCI trials [22]. The ADAS-Cog is sensitive to a wide range of disease severity specific to the central dysfunctions experienced in AD including memory, praxis, and language; it is regarded as the standard instrument for use in clinical trials as a primary index of cognitive change in AD [23]. The EXIT-25 and the MOCA provide a standardized clinical assessment of executive control functions relevant to SVCI [20, 21]. Though it is important to use clinically relevant measures, these generalized tests may not capture specific processes that may be impaired in mixed dementia. As such, additional tests of specific executive functions–i.e. working memory (Digits Forward and Backward Test), attention and response inhibition (Stroop Test), and set shifting (Trail Making Test)–may be more sensitive to subtle change [24].

The neurocognitive profile of SVCI with co-existing amyloid pathology remains to be elucidated. A better understanding of the cognitive dysfunctions associated with amyloid pathology in SVCI may be a useful adjunct in the clinical assessment of mixed SVCI-AD dementia. Thus, the purpose of this study was to understand whether individual differences in amyloid pathology might explain variations in cognitive impairment among individuals with clinical SVCI, using a clinically relevant neuropsychological test battery that is sensitive to both pathologies.

## Methods

### Study design and participants

We conducted a cross-sectional analysis of baseline data acquired from a proof-of-concept randomized controlled trial of aerobic exercise (i.e., NCT01027858) [25].

This study consisted of adults with a clinical diagnosis of mild SVCI. We recruited from the University of British Columbia Hospital Clinic for AD and Related Disorders, the Vancouver General Hospital Stroke Prevention Clinic, and specialized geriatric clinics in Metro Vancouver, BC. Clinical diagnosis of SVCI was made by neurologists and geriatricians based on the presence of both small vessel ischemic disease and cognitive syndrome. Small vessel ischemic disease was defined as evidence of relevant cerebrovascular disease on MRI brain imaging that included: 1) Periventricular and deep WML: patchy areas of low attenuation or diffuse symmetrical areas of low attenuation with ill defined margins extending to the centrum semiovale, and at least one lacune; 2) Absence of cortical and or cortico-subcortical non-lacunar territorial infarcts and watershed infarcts, hemorrhages indicating large vessel disease, signs of normal pressure hydrocephalus, or other specific causes of WML (i.e. multiple sclerosis, leukodystrophies, sarcoidosis, and brain irradiation). Cognitive syndrome was defined as a MOCA score < 26/30 at baseline–the MOCA is a brief screening tool for mild cognitive impairment with high sensitivity and specificity [26]. Furthermore, study participants exhibited progressive cognitive decline (compared with previous level of cognitive function) as confirmed through medical records or caregiver/family member interviews. Overall, participants were generally functioning independently and living in the community with minimal assistance by family or caregiver. All participants underwent a physician assessment to confirm current health status and eligibility for the study. Ethical approval was obtained from the Vancouver Coastal Health Research Institute (V07-01160) and the University of British Columbia’s Clinical Research Ethics Board (H07-01160). All participants provided written informed consent.

Individuals were eligible for study entry if they met the following criteria: 1) aged 55 years or older; 2) MOCA score < 26/30 at screening [26]; 3) Mini-Mental State Examination score ≥ 20 at screening [27]; 4) lived in Metro Vancouver, Canada and was able to read, write, and speak English; 5) if participants are on cognitive medications (i.e. donepezil, galantamine, rivastigmine, memantine, etc.) they must be on a fixed dose for the duration of the trial; 6) must be in sufficient health to participate in the study’s aerobic-based exercise training program; and 7) provide informed consent. Exclusion criteria included: 1) absence of small vessel ischemic lesions such as WML or lacunes on brain CT or MRI; 2) diagnosed with another type of dementia (e.g. AD, dementia with lewy bodies, or frontal-temporal dementia) or other neurological conditions (e.g. multiple sclerosis or Parkinson’s disease); 3) taking medications that may negatively affect cognitive function (e.g. anticholinergics); and 4) people who planned to participate in a clinical drug trial concurrent to this study. This analysis included a sub-set of 22 participants who met the overall study eligibility criteria and volunteered to complete a positron emission tomography (PET) scan.

### Descriptive variables

#### Demographic variables

Information regarding age, sex, education level, body mass index (BMI), and waist-hip ratio (WHR) was collected at study entry.

### WML quantification

#### Scanning protocol

Structural MRI data was acquired on a Philips 3 T Achieva MRI scanner (Philips Medical Systems, Best, The Netherlands) at the UBC MRI Research Centre. A T2-weighted scan and a Proton-Density-weighted (PD-weighted) scan were acquired. The repetition time (TR) and echo time (TE) for the T2-weighted images were TR = 5431 and TE = 90 ms and for the PD-weighted images were TR = 2000 ms and TE = 8 ms. Dimensions for the T2-and PD-weighted scans were 256 × 256 × 60 voxels with a voxel size of 0.937 × 0.937 × 3.000 mm.

#### Image analysis

Prior to lesion identification and segmentation, each MR image was preprocessed using standard and publicly available neuroimaging tools that included: 1) MR intensity inhomogeneity correction using a multiscale version of the nonparametric non-uniform intensity normalization method (N3) [28]; 2) a structure-preserving noise-removal filter (SUSAN) was applied [29]; and 3) all non-brain tissues were removed using the brain extraction tool (BET) [30].

WML were identified and digitally marked by a single radiologist with extensive experience in WML identification. The radiologist used the following guidelines in the seeding procedure, which was designed to be simple while enabling subsequent automated processing:

1. Mark all distinct WML regardless of size.
2. Place more than one point on a lesion if the additional points would help define the extent of the lesion.
3. Place at least one point near the center of each lesion.

WML were then segmented by a method that automatically computed the extent of each marked lesion [31]. This segmentation method has been extensively validated in large data sets with a large range of lesion loads, and was found to be highly accurate compared to radiologist segmentations and also robust to variations in the placement of the seed points [31]. Full details on the point placement procedure and subsequent automatic segmentation are described in previous work [31], but briefly, the seed points were processed by a customized Parzen windows classifier [32] to estimate the intensity distribution of the lesions. The algorithm included heuristics to optimize the accuracy of the estimated distributions by dynamically adjusting the position and the number of seed points used for the Parzen window computation, as well as a spatial method that approximated visual shape partitioning to identify areas that were likely to be false positives. The lesion masks were then used to quantify WML volumes in mm^3^.

### Dependent variables

#### Global cognitive function

##### MOCA

This is a cognitive screening tool that includes an assessment of set shifting, visuospatial abilities, short-term and working memory, attention, concentration, language, abstraction, and orientation to time and place–generally, it places emphasis on executive functions [33]. The MOCA computes a score out of 30 with lower scores indicating greater cognitive impairment. The MOCA is a sensitive tool for detecting both mild cognitive impairment [26] and VCI, including SVCI [34].

##### ADAS-Cog

This scale assesses memory, language, and praxis. There are 11 tests and scores range from 0 to 70 with higher scores indicating greater cognitive dysfunction. The inter-rater reliability of the ADAS-Cog is 0.989 and its test-retest reliability is 0.915 [35].

#### Executive functions

##### EXIT-2

This is a standardized clinical assessment of global executive functions [20, 36] and is designed to detect frontal systems pathology [37]. This test contains 25 items and scores range from 0 to 50 with higher scores indicating greater global executive dysfunctions. This measure can accurately separate non-demented subjects from those with cortical or subcortical dementias [38]. Its inter-rater reliability is 0.90 [39].

### Specific executive processes

Three specific executive processes were measured: 1) Working memory was assessed with the Verbal Digits Forward and Backward Tests [40]. Participants repeated progressively longer random number sequences in the same order as presented (forward) and in the reversed order (backward). The difference in score between the two tests was calculated, with smaller differences indicating better performance; 2) Selective attention and conflict resolution was assessed by the Stroop test [41], which involved three different conditions (80 trials each). First, participants read out words printed in black ink; second, they named the display colour of coloured-X’s; and third, they were shown a page with colour-words printed in incongruent coloured inks (e.g., the word “BLUE” printed in red ink). Participants were asked to name the ink colour in which the words were printed (while ignoring the word itself). We recorded the time participants took to read the items in each condition and calculated the time difference between the third condition and the second condition. Smaller time differences indicate better selective attention and conflict resolution; 3) Set shifting was assessed by the Trail Making Test (Part A and B) [42]. First, participants drew lines connecting encircled numbers sequentially (Part A) then they were asked to alternate between numbers and letters (Part B). The difference in time to complete Part B and Part A was calculated, smaller difference scores indicated better performance.

### Independent variables

#### Amyloid imaging

##### Scanning protocol

PET scans were performed using ^11^C-PIB produced at UBC TRIUMF. Scans were performed in 3-D mode using the GE Advance tomograph (General Electric, Canada/USA). Prior to injection, a 10-minute transmission scan with a ^68^Ge rod was collected for attenuation correction. After the transmission scan, 555 to 560 MBq of ^11^C-PIB was injected as a bolus into the antecubital vein and flushed with saline. A 90-minute dynamic acquisition started at tracer injection and data were framed into an 18×300 sec imaging sequence.

##### Image analysis

Parametric images of the non-displaceable binding potential (BP_ND_) [43] were generated using tissue input Logan graphical analysis [44, 45] with the cerebellum as the reference region. This method has been validated as reliable for quantification of amyloid deposition [46, 47]. A mean PIB-PET image, i.e. radiotracer concentration averaged over the entire scan duration was also formed for image co-registration and ROI definition purposes. Using SPM 8 (Wellcome Department of Cognitive Neurology, Institute of Neurology, University College London) each subject’s MRI image was co-registered to the corresponding mean PIB-PET image. Each subject’s MRI image was then normalized to the SPM MNI305 template and the corresponding transformation parameters were applied to the subject’s PET images (mean and parametric images). For those without MRI scans (5 subjects did not scan due to MR contraindications), the subject’s mean PIB-PET image was normalized to a mean PIB-PET image template in MNI space. This PIB-PET image template was created by averaging 6 healthy control PIB-PET scans that had all been warped with their own MRI to the SPM MNI305 template.

##### Regions of interest analysis

A custom set of regions of interest (ROIs) was defined on the coronal view of the MNI305 template [48]. These ROIs were transposed to each subject’s warped MRI and mean-PET images (in MNI space). ROIs were adjusted as necessary using both the MRI and mean PIB-PET image for guidance (1–2 pixels maximum). The modified set of ROIs was then applied to the parametric PIB-PET image and the average BP_ND_ within each ROI was extracted. Global PIB binding was determined by averaging values in bilateral frontal (combined orbitofrontal and medial prefrontal cortex), parietal (combined angular gyrus, superior parietal, precuneus, and supramarginal gyrus), temporal (combined lateral temporal and middle temporal gyrus), and occipital cortices, and anterior and posterior cingulate gyrus [49].

##### PIB-positive vs PIB-negative categorization

To determine PIB-positive/negative categorization we used standardized uptake values (SUV–tracer concentration/(injected dose/body weight)) normalized to the cerebellar cortex SUV, referred to as SUV ratio (SUVR–global SUV/cerebellar cortex SUV). An SUVR threshold of 1.50 was implemented–this PIB threshold was based on studies in a large group of cognitively normal subjects studied at the University of Pittsburgh [50] and is used by ADNI [51]. Participants with global SUVR above 1.50 were categorized as PIB-positive; participants with global SUVR below 1.50 were categorized as PIB-negative.

##### Amyloid and cognitive function

To determine the effect of amyloid on cognitive function we used Logan graphical analysis [44, 45] as it is more accurate when compared with SUVR [52].

### Covariates

#### APOE ε4 genotype

ApoE genotype was determined using TaqMan assay systems for the single nucleotide polymorphisms–219G/T. DNA was extracted from whole blood using an automated DNA extraction machine (AutogenflexStar, Autogen Inc, Hollisten, MA). Because the ApoE ε4 genotype is relatively rare, the ApoE ε4 genotype odds ratios was collapsed into 2 main categories: those with at least 1 ε4 allele and those with no ε4 allele [53].

### Statistical analysis

All statistical analyses were performed using Statistical Package for the Social Sciences 22.0. Initial data inspection determined that the distributions were normal (all skew values were less than the absolute value of 1). We first conducted Pearson bivariate correlations to determine which cognitive measures to include in our regression models–cognitive variables that were significantly correlated with PIB BP_ND_ values (p ≤ .05) were then entered in a hierarchical multiple linear regression analysis to determine the unique effect of amyloid on cognitive function. In the regression age, education, and ApoE ε4 status were entered in the first step as covariates, and PIB BP_ND_ was entered in the second step to determine the unique contribution of amyloid of cognitive function. However, due to our small sample size we conducted a regression analysis without covariates and a regression with covariates to ensure the robustness of our results. Also, we report adjusted *R*^2^ values, which penalizes the explained variance for each additional covariate. For each hierarchical regression model, we computed collinearity statistics (tolerance and variance inflation factor), histograms of the residuals, and scatterplots of the predicted versus residual values to ensure that the assumptions of linear regression were met. In all models, mutlicollinearity was not an issue among predictor variables, and the residuals were normally distributed and homoscedastic. These analyses also confirmed a linear association between the predictors and outcome variables for a hierarchical multiple linear regression.

## Results

### Descriptive variables

Twenty-two participants (8 females, 14 males) completed PIB-PET imaging. The mean age was 72.95 ± 7.76 years with an average MOCA score of 23.54 ± 2.34. Five out of 22 participants did not complete an MRI scan due to MR contraindications (e.g. presence of coronary stent or artificial optic lens). One scan was discarded from WML quantification analysis due to severe motion artifacts–with a subset of 16 participants, WML volume ranged from 23.49-3093.39 mm^3^ with an average of 616.41 ± 849.13 mm^3^. Of 22 participants, six were PIB-positive and 16 were PIB-negative. The global PIB BP_ND_ was 0.07 ± 0.23. Detailed demographic characteristics, neuropsychological test results, WML volume, and PIB BP_ND_ values are presented in Table 1. Compared with the total participants in the randomized controlled trial, this subset was not different in age (mean difference = 2.42, p > 0.05), but displayed higher MOCA scores (mean difference = 3.07, p < 0.01).

**Table 1:** Descriptive Characteristic

Variable	Mean	SD
Age	72.95	7.77
Female Sex, No. (%)	8 (36 %)	
Education, No. (%)		
High school education	1 (5 %)	
Trade or professional certificate or diploma	12 (55 %)	
University education	9 (41 %)	
MMSE (max. score 30)	27.50	1.95
MOCA (max. score 30)	23.55	2.34
WHR	0.91	0.08
BMI	26.95	4.78
PIB-positive, No. (%)	6 (27 %)	
Global PIB BP_ND_	0.07	0.23
WML volume (mm^3^), *n* = 16	616.41	849.13
Cognitive Assessments		
ADAS-Cog (max. score 70)	8.99	3.30
Exit-25 (max. score 50)	10.59	4.38
Stroop CW-C, sec.	61.44	26.01
Trails B-A, sec.	50.51	24.84
Digits F-B, sec.	3.23	2.79

*SD* = Standard Deviation, *MMSE* = Mini-Mental State Examination, *MOCA* = Montreal Cognitive Assessment, *WHR* = Waist-to-Hip Ratio, *BMI* = Body Mass Index, *ADAS*-*Cog* = Alzheimer’s Disease Assessment Scale – Cognitive subscale, *Exit*-25 = Executive Interview Test, *Stroop CW*-*W* = Stroop Color Words minus Stroop colored x’s, *Trails B*-*A* = Trails B (numbers and letters) minus Trails A (numbers), *Digits F*-*B* = Digits F-B = Digits Forwards minus Digits Backwards

### Bivariate correlations

PIB binding was significantly correlated with ADAS-Cog (r = 0.58, p < 0.01) and MOCA (r = −0.55, p < 0.01)–specifically, increased PIB BP_ND_ was associated with worse cognitive performance on the ADAS-Cog and the MOCA (Table 2). PIB BP_ND_ was not significantly correlated with the EXIT-25 or with any of the specific executive processes (p > 0.05).

**Table 2:** Correlation Matrix

	PIB BP_ND_	Age	Education	APOE ε4	ADAS-Cog	MOCA	EXIT 25	Digits	Stroop	Trails
PIB BP_ND_										
Age	−0.01									
Education	0.09	0.21								
APOE ε4	0.36	0.13	0.04							
ADAS-Cog	0.58**	0.15	0.04	0.61**						
MOCA	−0.55*	−0.12	−0.25	−0.16	−0.41					
EXIT-25	0.13	0.18	0.22	0.25	0.43*	−0.40				
Digits	0.02	0.12	0.34	−0.45*	−0.26	0.17	−0.04			
Stroop	0.17	−0.04	0.04	−0.11	0.34	−0.18	0.34	0.04		
Trails	0.19	−0.02	0.12	0.48*	0.28	−0.18	0.29	0.01	0.13	

*significant at *p* < 0.05; **significant at *p* < 0.01

### Linear regression

To determine the independent association between PIB BP_ND_ and ADAS-Cog and MOCA scores we conducted a hierarchical multiple linear regression.

### ADAS-Cog

Without covariates in the model, PIB rentention accounted for 31.0 % (adjusted R-square) of the variance in ADAS-Cog scores (F [1, 20] = 10.27, p = 0.00–Table 3). When controlled for age, education, and APOE ε4, this accounted for 27.0 % of the variance in ADAS-Cog scores. Adding PIB BP_ND_ to the model resulted in a significant adjusted R-square change of 15.0 % (F Change [1, 17] = 5.78, Sig F Change = 0.03–Table 3). The total adjusted variance accounted by the final model was 42.0 %.

**Table 3:** Multiple linear regression models assessing the contribution of PIB retention on ADAS-Cog

Independent variables	R^2^	Adjusted R^2^	R^2^ Change	Unstandardized B (Standard error)	Standardized β	*P*- Value
Model 1						
PIB BP_ND_	0.34	0.31	-	8.56 (2.67)	0.58	0.00
Model 2						
Step 1	0.37	0.27	0.37*			
Age				0.03 (0.08)	0.070	0.73
Education				0.02 (0.68)	0.01	0.97
APOE ε4				4.14 (1.30)	0.60	0.01
Step 2	0.53	0.42	0.16*			
Age				0.04 (0.07)	0.10	0.56
Education				−0.11 (0.61)	−0.03	0.86
APOE ε4				3.07 (1.24)	0.44	0.02
PIB BP_ND_				6.31 (2.62)	0.43	0.03

*significant at *p* < 0.05

### MOCA

Without covariates in the model, PIB retention accounted for 27.0 % (adjusted R-square) of the variance in MOCA scores (F [1, 20] = 8.66, p = 0.01–Table 4). When controlled for age, education, and APOE ε4 this accounted for–7.0 % of the variance in MOCA scores, which suggests that the penalty for adding these covariates outweighed their explained variance in MOCA scores. Adding PIB BP_ND_ to the model resulted in a significant adjusted R-square change of 27.0 % (F Change [1, 17] = 6.98, Sig F Change = 0.02–Table 4). The total adjusted variance accounted by the final model was 20.0 %.

**Table 4:** Multiple linear regression models assessing the contribution of PIB retention on MOCA

Independent variables	R^2^	Adjusted R^2^	R^2^ Change	Unstandardized B (Standard error)	Standardized β	*P*- Value
Model 1						
PIB BP_ND_	0.30	0.27	-	−5.75 (1.95)	−0.55	0.01
Model 2						
Step 1	0.09	−0.07	0.09			
Age				−0.02 (0.07)	−0.06	0.81
Education				−0.58 (0.59)	−0.23	0.34
APOE ε4				−0.72 (1.12)	−0.15	0.53
Step 2	0.35	0.20	0.27*			
Age				−0.03 (0.06)	−0.10	0.63
Education				−0.46 (0.51)	−0.18	0.39
APOE ε4				0.27 (1.04)	0.06	0.80
PIB BP_ND_				−5.80 (2.20)	−0.56	0.02

*significant at *p* < 0.05

## Discussion

To date, few studies have focused on the role of co-existing amyloid pathology in a primary SVCI diagnosis. Our study found that six out of twenty-two participants with clinical SVCI were PIB-positive. In assessing the effect of amyloid on cognitive function, we found that increased global amyloid deposition–suggestive of co-existing Alzheimer pathology–was significantly associated with worse cognitive function as indicated by the ADAS-Cog and MOCA. Our findings concur with and extend the results of previous literature assessing the role of amyloid on cognitive function in people with mild cognitive impairments (MCI) and healthy older adults.

The ADAS-Cog primarily assesses episodic memory and has been linked to amyloid deposition [54]. This association is present in both healthy older adults and people with MCI. Longitudinal studies in healthy older adults found that increased PIB binding was associated with greater memory decline over time [55, 56] and may be indicative of preclinical AD [56]. A similar association is found in people with MCI. Several studies have found increased PIB binding to be strongly correlated with episodic memory impairments in amnestic MCI subtypes [57, 58]; furthermore, PIB-positive amnestic MCI patients are more likely to progress to AD [59, 60]. Together, these previous studies have established the association between amyloid and memory impairments within an AD context. The current study extends previous knowledge of amyloid deposition by showing that greater amyloid deposition on PIB-PET screening is associated with greater memory impairment in a SVCI cohort.

Our study also found increased amyloid to be associated with lower MOCA scores, which assesses a mix of cognitive functions with an emphasis on executive functions. Though AD is typically associated with memory impairments, people in the early stages of AD display executive dysfunctions [61, 62]; thus, it is plausible that amyloid deposition would also be associated with executive dysfunctions. However, we note that we did not find a significant association with specific executive measures (i.e., Digit Span Test, Stroop test, and Trail Making Test) and the EXIT-25 test. No other studies have reported data on the EXIT-25 and few studies have examined the effect of amyloid deposition on specific executive processes. One reason for these non-significant results may lie in the minimal power of these tests to detect an effect. A complex statistical study conducted by ADNI found a composite score (ADNI-EF included: Category Fluency, Clock Drawing, WAIS-R Digit Symbol, Digit Span Backwards, and the Trail Making Test including Trails A, Trails B, and Trails B minus Trails A) to be superior to any independent measure of executive functioning. Specifically, ADNI-EF was sensitive to capturing changes in cognitive function over time and was the strongest baseline predictor of conversion to AD [63]. Although the MOCA does include a memory subtest, it places greater emphasis on tasks of executive function and has similar components to ADNI-EF (includes Clock Drawing, Digit Span Backwards, and Trail Making and additionally includes a phonemic fluency task, a two-item verbal abstraction task, a sustained attention task, and a concentration task). Thus, the MOCA–as a global composite measure–may be more sensitive compared with specific executive processes.

Overall, we found that amyloid was associated with impairments in multiple domains of cognitive function including memory and executive dysfunctions in people with clinical SVCI. A similar study conducted by Lee and colleagues [64] found that PIB retention in people with small vessel MCI was associated with impairments in multiple domains of cognitive function including language, visuospatial, memory, and executive functions. Furthermore, these results concur with a study published by Nordlund and colleagues [65] who found that people with cognitive impairments in multiple domains (i.e. memory and executive dysfunctions) were more likely to convert to mixed dementia and vascular dementia compared with people who exhibited either memory or executive dysfunction alone.

However, our conclusions are not without limitations. First, this is an exploratory study, and thus, is limited by its small sample size; therefore, we are limited in our ability to detect smaller effects and future studies are required to confirm and extend our current results. We also did not account for the effect of NFT on cognitive function. This is of particular importance as neocortical NFT is more consistently correlated with dementia severity, and it is suggested that NFT may mediate the association of amyloid on cognitive function [66]. In addition, we were not able to acquire MRI scans in all participants and did not have the sample size to include WML as a covariate. As a result, it is not clear how WML may have uniquely contributed to performance on the ADAS-Cog and MOCA. This is particularly important as declines in memory and executive functions have been linked to increased subcortical white matter disease [67–69]. However, a study published by Park and colleagues [49] investigating the relationship between cerebrovascular disease, amyloid, and cognitive function in SVCI suggested that amyloid burden and SVCI pathology were largely unrelated and that the effects of amyloid on cognition is independent of markers of SVCI pathology. The unique impact of amyloid on cognitive function is further supported by evidence in cerebrospinal fluid (CSF). A study assessing the role of amyloid beta (the 42-amino-acid form–Aβ_1–42_) and neurofilament light (NF-L)–elevated concentration of NF-L in CSF are associated with WML and small vessel disease—in CSF found that only Aβ_1–42_ was associated with worse cognitive outcomes in people with cerebral vascular disease [70]. Overall, the results of our study and previous studies [64, 70, 71] suggest that increased amyloid is independently associated with worse cognitive outcomes in people with vascular disease.

## Conclusion

It is reported that approximately 33 % of those with SVCI exhibit amyloid pathology [71]. Yet, few studies have investigated the effect of amyloid deposition in people with SVCI and fewer studies have assessed PIB rentention as a continuous variable. Categorical diagnostic classifications of AD, SVCI, and mixed AD/SVCI in clinical practice fail to take into account the reality of a gradient of brain amyloid deposition across these disease states. Our findings point to the important role of amyloid deposition for cognitive function even among those with a primary SVCI diagnosis. As such, therapeutic approaches targeting SVCI must consider the potential role of amyloid for the optimal care of those with mixed dementia.
