# Erratum to: Exploring the effects of coexisting amyloid in subcortical vascular cognitive impairment

**DOI:** 10.1186/s12883-016-0673-5

**Published:** 2016-08-31

**Authors:** Elizabeth Dao, Ging-Yuek Robin Hsiung, Vesna Sossi, Claudia Jacova, Roger Tam, Katie Dinelle, John R. Best, Teresa Liu-Ambrose

**Affiliations:** 1 Djavad Mowafaghian Centre for Brain Health, University of British Columbia, 2215 Wesbrook Mall, Vancouver, BC V6S 0A9 Canada; 2 Department of Medicine, University of British Columbia, UBC Hospital S152, 2211 Wesbrook Mall, Vancouver, BC V6T 2B5 Canada; 3 Department of Physics and Astronomy, University of British Columbia, 6224 Agricultural Road, Vancouver, BC V6T 1Z1 Canada; 4 School of Professional Psychology, Pacific University, 190 SE 8th Avenue, Hillsboro, OR 97123 USA; 5 Department of Radiology, University of British Columbia, 3350-950W 10th Avenue, Vancouver, BC V5Z 1M9 Canada; 6 MS/MRI Research Group, University of British Columbia, 2215 Wesbrook Mall, Vancouver, BC V6S 0A9 Canada; 7 PET Group, Vancouver Hospital and Health Science Centre, Brain Research Centre, 2211 Westboork Mall, Vancouver, BC V6T 2B5 Canada; 8 Department of Physical Therapy, University of British Columbia, 212-2177 Wesbrook Mall, Vancouver, BC V6T 1Z3 Canada

## Erratum

After publication of the original article [1], it came to the authors’ attention that there were data entry errors in the following variables: age, sex, education, MOCA, WML volume, and ADAS-Cog. Changes have been made to Tables 1, 2, 3 and 4, to reflect the updated results, which are published in their correct version here. However, the authors note that the study conclusions have not changed.

**Table 1:** Descriptive characteristic

Variable	Mean	SD
Age	71.73	7.91
Female Sex, No. (%)	7	32
Education, No. (%)		
High school education	6	27
Trade or professional certificate or diploma	2	9
University education	14	64
MMSE (max. score 30)	27.50	1.95
MOCA (max. score 30)	23.32	2.08
WHR	0.91	0.08
BMI	26.95	4.78
PIB-positive, No. (%)	6	27.27
Global PIB BP_ND_	0.07	0.23
WML volume (mm^3^), *n* = 16	2004.40	2761.15
Cognitive Assessments		
ADAS-Cog (max. score 70)	9.63	3.88
Exit-25 (max. score 50)	10.59	4.38
Stroop CW-C, sec.	61.44	26.01
Trails B-A, sec.	50.51	24.84
Digits F-B, sec.	3.23	2.79

*SD* Standard Deviation, *MMSE* Mini-Mental State Examination, *MOCA* Montreal Cognitive Assessment; *WHR* Waist-to-Hip Ratio, *BMI* Body Mass Index, *ADAS-Cog* Alzheimer’s Disease Assessment Scale – Cognitive subscale, *Exit-25* Executive Interview Test, *Stroop CW-W* Stroop Color Words minus Stroop colored x’s, *Trails B-A* Trails B (numbers and letters) minus Trails A (numbers), *Digits F-B* Digits Forwards minus Digits Backwards

**Table 2:** Correlation matrix

	PIB BP_ND_	Age	Education	APOE ε4	ADAS-Cog	MOCA	EXIT 25	Digits	Stroop	Trails
PIB BP_ND_										
Age	-0.00									
Education	-0.23	-0.39								
APOE ε4	0.36	0.09	-0.20							
ADAS-Cog	0.53*	0.16	-0.11	0.53*						
MOCA	-0.54*	-0.20	0.06	-0.11	-0.45*					
EXIT-25	0.13	0.20	-0.20	0.25	0.49*	-0.52*				
Digits	0.02	0.17	-0.35	-0.45*	-0.30	0.13	-0.04			
Stroop	0.17	-0.06	0.02	-0.11	0.27	-0.29	0.34	0.04		
Trails	0.19	-0.08	-0.25	0.48*	0.15	-0.18	0.29	0.01	0.13	

*significant at *p* ≤ 0.05

**Table 3:** Multiple linear regression models assessing the contribution of PIB retention on ADAS-Cog

Independent variables	R^2^	Adjusted R^2^	R^2^ Change	Unstandardized B (Standard Error)	Standardized β	*P*- Value
Model 1						
PIB BP_ND_	0.28	0.24	-	9.09 (3.29)	0.53	0.01
Model 2						
Step 1	0.30	0.18	0.30			
Age				0.07 (0.11)	0.14	0.54
Education				0.41 (1.86)	0.05	0.83
APOE ε4				4.30 (1.64)	0.53	0.02
Step 2	0.44	0.31	0.15*			
Age				0.09 (0.10)	0.18	0.37
Education				1.15 (1.74)	0.14	0.52
APOE ε4				3.20 (1.59)	0.39	0.06
PIB BP_ND_				7.23 (3.42)	0.42	0.05

*significant at *p* ≤ 0.05

**Table 4:** Multiple linear regression models assessing the contribution of PIB retention on MOCA

Independent variables	R^2^	Adjusted R^2^	R^2^ Change	Unstandardized B (Standard Error)	Standardized β	*P*- Value
Model 1						
PIB BP_ND_	0.29	0.26	-	-5.00 (1.74)	-0.54	0.01
Model 2						
Step 1	0.05	-0.11	0.05			
Age				-0.06 (0.07)	-0.21	0.41
Education				-0.22 (1.16)	-0.05	0.85
APOE ε4				-0.43 (1.02)	-0.10	0.68
Step 2	0.37	0.22	0.32*			
Age				-0.07 (0.06)	-0.28	0.20
Education				-0.80 (0.99)	-0.18	0.43
APOE ε4				0.44 (0.91)	0.10	0.63
PIB BP_ND_				-5.71 (1.95)	-0.62	0.01

*significant at *p* ≤ 0.05
